# Editorial: Biomarkers of peripheral and central auditory system integrity and function

**DOI:** 10.3389/fneur.2024.1374844

**Published:** 2024-02-05

**Authors:** Stefan Weder, Christo William Bester, Stephen O'Leary

**Affiliations:** ^1^Department of Otorhinolaryngology, Head and Neck Surgery, Inselspital, Bern University Hospital, Bern, Switzerland; ^2^Department of Otolaryngology, The University of Melbourne, Melbourne, VIC, Australia

**Keywords:** auditory system, diagnostic and therapeutic applications, patient-centered approaches, biomarkers, technological advances

Objective biomarkers of auditory system integrity and function are becoming increasingly indispensable in the diagnostic, therapeutic, and rehabilitative aspects of hearing impairments. This Research Topic compiles pioneering studies on such biomarkers and measurement methods. Ranging from sophisticated genetic sequencing to advanced electrophysiological recordings and imaging methods. These techniques aim to enhance our understanding of auditory pathways, improve therapeutic indications, and provide refined patient counseling and monitoring throughout treatment.

Here, we present 10 studies that epitomize the progress in this field:

- The study by Sun et al. on the genetic etiology of Auditory Neuropathy Spectrum Disorder offers significant insights into the disorder's genetic diversity, paving the way for personalized therapy.
- Mushtaq et al.'s work demonstrates the potential for cochlear implant users to self-assess cochlear health, indicating a shift toward more autonomous patient monitoring.
- Han et al. investigate the unique cortical activation patterns in individuals with single-sided deafness, enhancing our understanding of speech processing in challenging environments.
- The research by Caldas et al. evaluates a novel assessment tool for cochlear implants, proposing enhancements in cochlear implant fittings' accuracy.
- Haggerty et al. focus on the complexities of cochlear synaptopathy, advocating for sophisticated diagnostic methods.
- The study by Chen et al. highlights the crucial impact of treatment timing on sudden sensorineural hearing loss recovery, emphasizing rapid intervention.
- Schuerch et al. employ deep learning algorithms to improve the objectivity and reliability of intracochlear electrocochleography.
- Schraivogel et al. reveal the potential of impedance subcomponents in cochlear implants as specific biomarkers for residual hearing.
- Kadowaki et al. present an objective measure for auditory temporal resolution, paving the way for improved assessments of auditory processing.
- The comparative study by Gawliczek et al. on Auditory Brainstem Response and Extracochlear Electrocochleography in evaluating coupling efficiency in middle ear implant surgery offers valuable normative data for surgical precision.

## Conclusion

The studies featured in this Research Topic highlight the importance of objective biomarkers of auditory system integrity and function in enhancing the diagnosis, treatment indications, and monitoring of hearing impairments. These findings advocate for nuanced, patient-centered approaches and emphasize the significance of integrating these biomarkers into clinical practice. The continued exploration and application of these objective measures promise to enhance the lives of individuals with hearing impairments and deepen our overall understanding of the auditory system.

## Author contributions

SW: Writing—original draft. CB: Writing—review & editing. SO'L: Writing—review & editing.

